# Chicoric acid is a potent anti-atherosclerotic ingredient by anti-oxidant action and anti-inflammation capacity

**DOI:** 10.18632/oncotarget.16768

**Published:** 2017-03-31

**Authors:** Kun-Ling Tsai, Chung-Lan Kao, Ching-Hsia Hung, Yung-Hsin Cheng, Huei-Chen Lin, Pei-Ming Chu

**Affiliations:** ^1^ Department of Physical Therapy, College of Medicine, National Cheng Kung University, Tainan, Taiwan; ^2^ Department of Physical Medicine and Rehabilitation, Taipei Veterans General Hospital, Taipei, Taiwan; ^3^ Institute of Allied Health Sciences, College of Medicine, National Cheng Kung University, Tainan, Taiwan; ^4^ Department of Education and Research, Taipei City Hospital, Taipei, Taiwan; ^5^ Department of Physical Therapy, Shu-Zen Junior College Of Medicine And Management, Kaohsiung, Taiwan; ^6^ Department of Anatomy, School of Medicine, China Medical University, Taichung, Taiwan

**Keywords:** atherosclerosis, free radical, antioxidant, chicoric acid, inflammation, Gerotarget

## Abstract

Atherosclerotic cardiovascular disease is linked to both oxidative stress and endothelial cell dysfunction. Chicoric acid has antioxidant and anti-inflammatory properties. In the present investigation, we demonstrated that chicoric acid inhibits oxidized low-density lipoprotein (oxLDL)-facilitated dysfunction in human umbilical vein endothelial cells (HUVECs). Oxidative injuries were tested by investigating the formation of intracellular reactive oxygen species (ROS) and by examining the activity of antioxidant enzymes and the function of endothelial nitric oxide synthase (eNOS). We also confirmed that chicoric acid mitigates apoptotic features caused by oxLDL, such as the subsequent break down of mitochondrial transmembrane potential and the activation of Bax, which promote DNA strand breaks and activate caspase-3. Moreover, our data revealed that chicoric acid attenuated the oxLDL activation of NF-?B, the attachment of THP-1 cells and the overexpression of adhesion molecules in human endothelial cells. The results of this study suggest a potential molecular mechanism through which chicoric acid inhibits oxLDL-induced human endothelial dysfunction.

## INTRODUCTION

Atherosclerotic damage is linked to oxidative injuries [1]. The initial stages of atherosclerosis are facilitated by the augmentation of oxidized low-density lipoprotein (oxLDL) and the aggregation of vascular cells. The subsequent overexpression of adhesion molecules and enhanced adherence of monocytes to endothelial cells were shown as important events in the progression of atherosclerosis [2, 3]. The oxLDL-induced endothelial toxicity and morphological changes observed in cultured endothelial cells are alike to those detected in animal in endothelial cells overlying atherosclerotic areas [4].

oxLDL-induced reactive oxygen species (ROS) formation plays a vital role in the mediation of endothelial dysfunction and thus regulate endothelial apoptosis as well as inflammation [5, 6]. High doses of ROS can cause cell dysfunction and death *via* the oxidation-induced modification of proteins, nucleic acids, carbohydrates and lipids. Those modifications consequently regulate endothelial inflammatory responses and apoptotic events [7]. Moreover, these pro-apoptotic responses are related to disturbances in mitochondrial membrane permeability and the subsequent release of cytochrome c, and the up-regulation of executioner caspases [8]. The presence of activated NF-κB in atherosclerotic lesion suggests a potential role of NF-κB in atherosclerotic pathology; a local inflammatory even is associated in atherosclerotic pathology, and NF-κB is shown to be involved in this process [9–11]. NF-κB also mediates the function of various genes involved in the balance between cell survival and apoptosis [12]. E- and P-selectin, vascular cell adhesion molecule-1 (VCAM-1) and intercellular cell adhesion molecule-1 (ICAM-1) are adhesion molecules in human endothelial cells. Moreover, NF-κB plays a positive role in the activation of those adhesion molecules. In addition, adhesion molecules are thought to be early indicators of atherosclerosis [13]. For this reason, therapeutic strategies involving inhibitors of oxLDL-mediated endothelial dysfunction may reduce the progression of atherosclerotic pathology, inhibit their morbidity and promote the survival of patients with cardiovascular diseases.

Chicoric acid is one compound which could be obtained from isolated and purified plant and vegetables. In addition, chicoric acid has been reported as one daily nutraceutical to enhance antioxidant activity [14]. Tousch et al suggested chicoric acid is one novel ingredient able to increase insulin release as well as glucose uptake. Chicoric acid was also shown to facilitate 3T3-L1preadipocytes dysfunction by modulation of ROS-mediated PI-3K/AKT and MAPK mechanism [15]. Therefore, chicoric acid is described as having potential anti-diabetic and anti-obesity capacities. Landmann et al reported chicoric acid mitigates acute alcohol-induced hepatic steatosis in mice through oxidative stress inhibition and anti-inflammation properties [16]. Liu et al reported that chicoric acid intervention protects against systemic inflammation-caused memory impairment and amyloidogenesis through inactivation of NF-κB, indicating that chicoric acid has positive effects in management of chronic systemic diseases [17]. In contrast to the previously mentioned studies of the *in vivo* and *in vitro* antioxidant and anti-inflammatory effects of chicoric acid, no investigations were performed on chicoric acid effects on oxLDL-caused oxidative injuries in human endothelial cells.

Therefore, in this study, we explored whether chicoric acid inhibits oxLDL-mediated endothelial injuries and attempted to prove the cytoprotective effects of Chicoric acid, which prevent the oxLDL-mediated inhibition of endothelial NO synthase (eNOS), the overexpression of oxLDL-induced adhesion molecules and the monocyte adhesion. Additionally, we also investigated several apoptotic events, including mitochondrial destabilization and the up-regulation of caspases.

## RESULTS

### Chicoric acid reduces oxLDL-facilitated ROS generation in HUVECs

**Figure 1:**
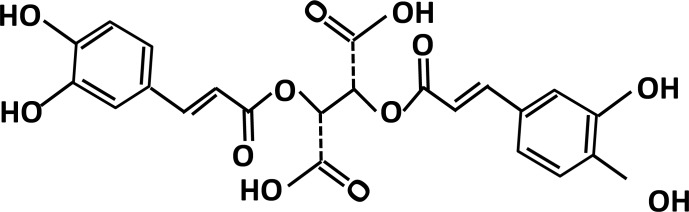
The chemical structure of chicoric acid

**Figure 2:**
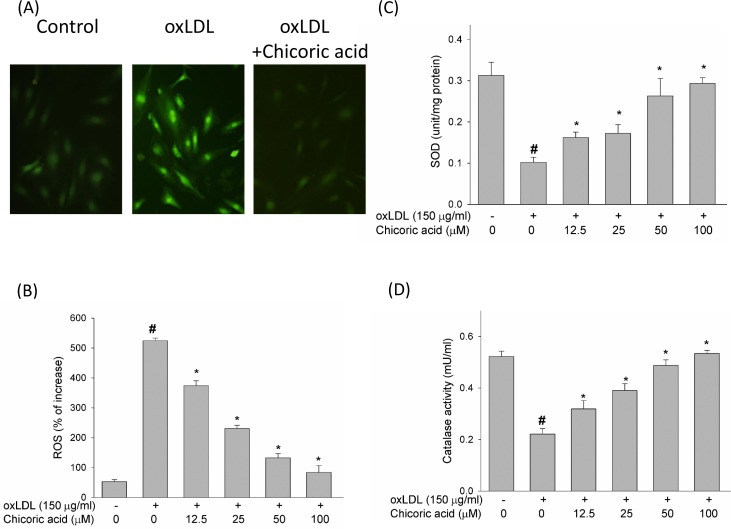
The inhibitory effects of chicoric acid on oxLDL-facilitated ROS generation in endothelial cells After pretreatment for 2 h with the indicated concentrations of chicoric acid (12.5-100 μM), HUVECs were incubated with the H_2_O_2_-sensitive fluorescent probe DCF-AM (10 μM) for 1 h, followed by treatment with 150 μg/ml oxLDL. **A**. Fluorescence images revealed the ROS level in control cells (left) and HUVECs stimulated with oxLDL (middle) in the presence of 100 μM chicoric acid (right). **B**. Fluorescence intensity of HUVECs was measured with a fluorescence microplate reader. Fluorescence distribution of DCF-AM oxidation was expressed as a percentage of increased intensity. The activity of **C**. SOD and **D**. catalase in HUVECs stimulated with oxLDL in the absence or presence of indicated concentrations of chicoric acid were determined. Data are expressed as the mean ± SD. of three independent analyses. # *P* < 0.05 *vs*. untreated control; *P < 0.05 compared with oxLDL treatment.

ROS act a key role in regulation of pro-apoptotic signal transduction pathways and cell death [18]. Therefore, we initially studied the effects of chicoric acid on the formation of ROS, a potential candidate related to oxLDL-promoted endothelial dysfunction, by DCF-AM. The chemical structure of chicoric acid was revealed in Figure 1. Pretreatment of endothelial cells with chicoric acid (12.5-100 μM) for 2 h before stimulate with oxLDL inhibited the formation of ROS (all P < 0.05) (Figure 2A, 2B). Moreover, ROS are known to attenuate antioxidant enzymes, promoting an imbalance that favors oxidative stress [19]. Therefore, we tested the activity of both superoxide dismutase (SOD) and catalase in oxLDL-exposed endothelial cells [5]. Our results revealed that oxLDL induced ROS formation but not inhibited SOD and catalase activity in 2h treatment. However, oxLDL reduced SOD and catalase activity in 24h treatment (data not show). This result suggested that oxLDL-induced ROS formation may the up-stream signaling in regulation of cell death and antioxidant enzymes dysfunction. However, In Figure 2C and 2D, we show that endothelial SOD and catalase activity was significantly reduced after exposure to oxLDL. These results were effectively reversed by chicoric acid treatment.

### Chicoric acid reverses oxLDL-inhibited eNOS phosphorylation

**Figure 3:**
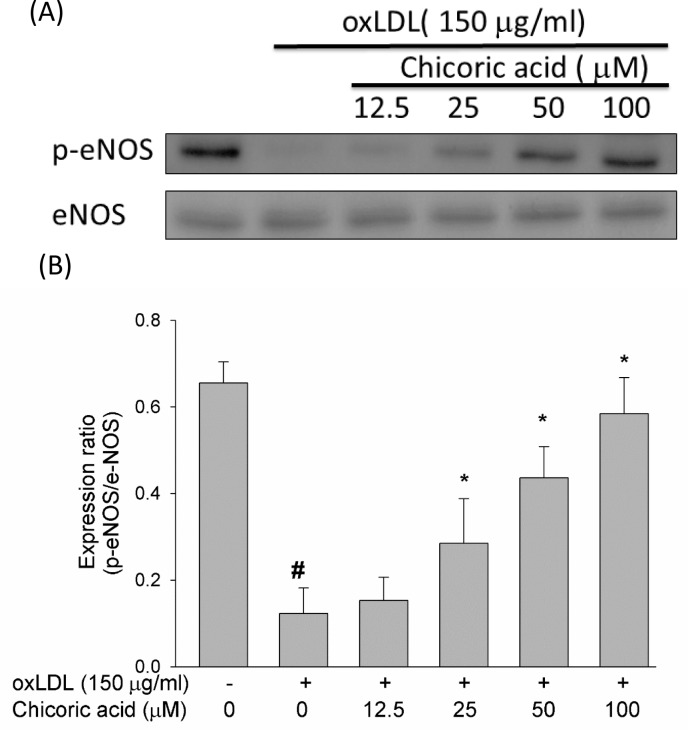
Effects of chicoric acid on oxLDL-repressed eNOS protein phosphorylation HUVECs were pretreated for 2 hours with the indicated concentrations of chicoric acid (12.5-100 μM), followed by oxLDL for 24 hours. **A**.,**B**. For Western blot analyses, a monoclonal anti-p-eNOS (Ser1177) and a monoclonal anti-eNOS antibody (for normalization) were used. The values represent means ± SD error from three separate experiments. # *P* < 0.05 *vs*. untreated control; *P < 0.05 compared with oxLDL treatment.

A previous report suggested that the secretion of NO from HUVECs is inhibited by oxLDL, facilitating cell death in human endothelial cells [20]. However, whether chicoric acid protects endothelial cells from oxLDL-inhibited eNOS phosphorylation remained unknown. Therefore, we tested the effects of chicoric acid on eNOS phosphorylation. As shown in Figure 3A and 3B, oxLDL largely inhibited eNOS protein phosphorylation in endothelial cells after 24 h of stimulation; this result was significantly reversed by chicoric acid intervention.

### Effects of chicoric acid on oxLDL-promoted apoptosis

**Figure 4:**
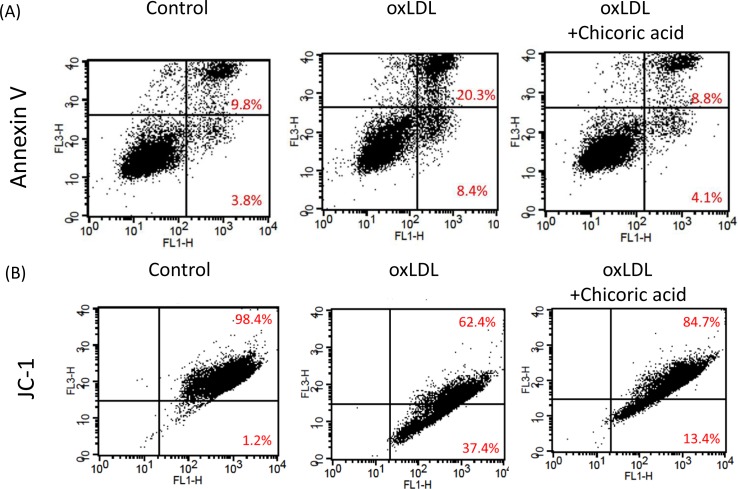
Chicoric acid blocked oxLDL-induced apoptosis in HUVECs **A**. HUVECs were incubated with oxLDL (150 μg/ml) in the absence or presence of indicated concentrations of chicoric acid for 24 h. Apoptotic and necrotic death of oxLDL-exposed endothelial cells were tested by a FITC-labeled annexin V assay and PI staining. Flow cytometry was used for confirmation. **B**. ΔΨm was inspected with the signal from monomeric and J-aggregate JC-1 fluorescence, as described in the Materials and Methods. (left) No treatment; (middle) oxLDL; (right) oxLDL + chicoric acid. JC-1 fluorescence was confirmed by flow cytometry.

Annexin V was used to determine both the pro-apoptotic effects of oxLDL and the anti-apoptotic ability of chicoric acid on endothelial cells. Flow cytometric analysis demonstrated that apoptotic cells (20.3%) and necrotic cells (8.4%) were found in endothelial cells stimulated with oxLDL. The apoptotic cells in HUVECs pretreated with 100 μM chicoric acid (8.8%) was similar to that observed in control sample (Figure 4A). Moreover, the opening of the mitochondrial permeability transition pore (PTP) has been suggested to play a vital role in the pathway that promotes apoptosis. The mitochondrial membrane potential (Ψm) collapses by both the disability of the electrochemical gradient caused *via* pore opening and the rupture of the outer mitochondrial membrane [21]. We subsequently investigated mitochondrial permeability to determine whether chicoric acid inhibits mitochondrial stability after stimulate with oxLDL. As shown in Figure 4B, oxLDL depolarized the mitochondrial membrane in endothelial cells, as demonstrated by the up-regulation in FL1 (middle panel), Intervention with chicoric acid resulted in stability of the mitochondrial transmembrane potential, as indicated by both the reduction of FL1 and the reversion n of FL2 (right panel).

**Figure 5:**
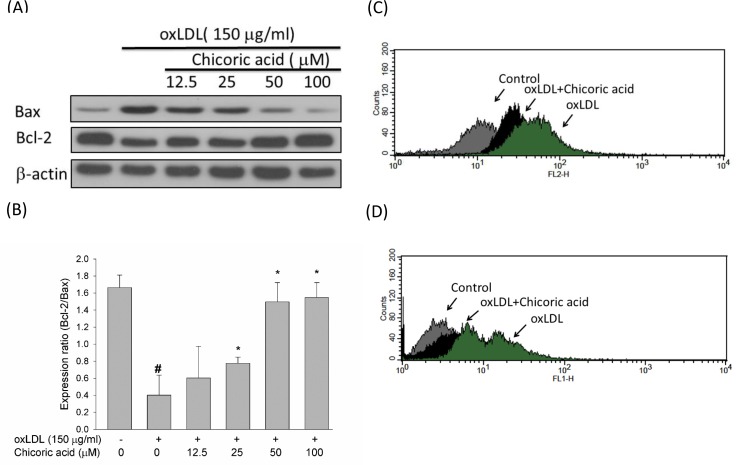
Effects of chicoric acid on oxLDL-triggered endothelial apoptosis **A**. Immunoblotting analysis of apoptotic cells in HUVECs exposed to oxLDL and chicoric acid. HUVECs were incubated with 150 μg/ml oxLDL in the absence or presence of indicated concentrations (12.5-100 μM) of chicoric acid for 24 h. Representative Western blots and summary data showing that oxLDL up-regulated Bax and down-regulated Bcl-2 proteins. Intervention with chicoric acid suppressed the above mentioned oxLDL-induced responses. Results were confirmed by densitometric analysis **B**.; the values are presented as means ± SD of three separate experiments. #*P*< 0.05 *vs*. untreated control; *P < 0.05 *vs*. oxLDL treatment. **C**. Effect of chicoric acid on oxLDL-induced caspase-3 activation. HUVECs were incubated with oxLDL in the absence (right) and presence (middle) of 100 μM chicoric acid. Activated of caspase-3 was examined by using flow cytometry (control: grey; oxLDL: black; oxLDL with chicoric acid: green). **D**. Effect of chicoric acid on oxLDL-induced DNA damage. HUVECs were incubated with oxLDL in the absence (right) and presence (middle) of 100 μM chicoric acid. TUNEL assay was examined by using flow cytometry (control: grey; oxLDL: black; oxLDL with chicoric acid: green).

Anti-apoptotic genes in the Bcl-2 family mediate mitochondrial outermembrane permeability and able to act as either an anti-apoptotic c or a pro-apoptotic regulator. The activation of the pro-apoptotic protein p53 is thought to be an important process in oxLDL-induced endothelial cell death *via* the promotion of ROS generation and subsequent induction of a conformational change in Bax that empowers the mitochondrial translocation of pro-apoptotic events [22]. We used a Western blotting assay to test the protective effects of chicoric acid against the oxLDL-mediated activation of Bax and the inhibition of Bcl-2. In Figure 5A, 5B, we confirmed that chicoric acid both significantly mitigated the activation of Bax and reversed the expression of Bcl-2. Caspase-3 also acts a vital role in mitochondrial apoptosis [21]. To test whether chicoric acid inhibits oxLDL-activated caspase-3 activation, we validated the active form of caspase-3 using a flow cytometry assay. In Figure 5C, the activity of caspase-3 enriched in endothelial cells exposed to oxLDL. However, the oxLDL-activated caspase-3 was mitigated in cells pretreated with 100 μM chicoric acid. In order to determine whether that oxLDL-caused cell death is an apoptotic response, oxLDL-treated cells were analyzed biochemically *via* flow cytometry using a TUNEL assay. As shown in Figure 5D, the results of indicated that chicoric acid is a potent repressor of oxLDL-caused cytotoxicity in HUVECs.

### Chicoric acid reduces the oxLDL-induced cytotoxicity of endothelial cells

**Figure 6:**
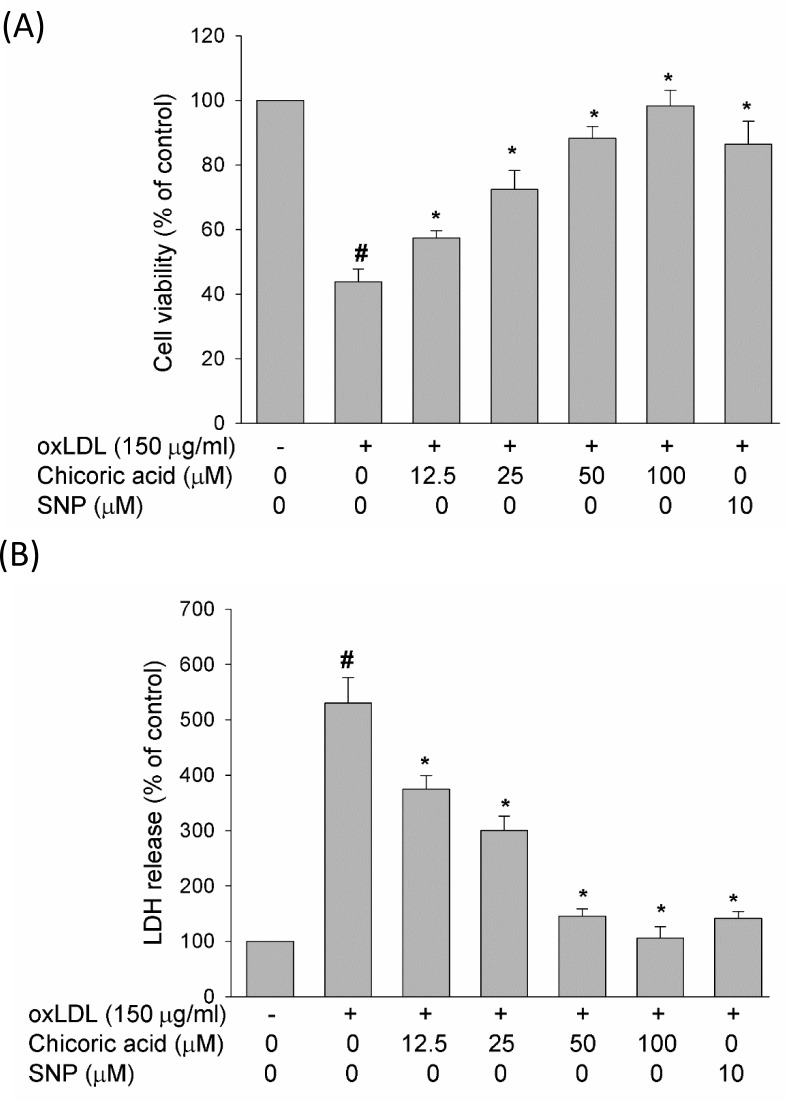
Effect of chicoric acid on oxLDL-reduced cell viability HUVECs were incubated with oxLDL (150 μg/ml) in the absence and presence of indicated concentrations of chicoric acid or SNP (NO donor). **A**. Viability was determined via MTT assay **B**. and LDH release. The values represent means ± SD from three separate experiments. #*P* < 0.05 *vs*. untreated control; *P < 0.05 *vs*. oxLDL treatment.

At the end of stimulation with oxLDL, the viability of HUVECs exposed with oxLDL in the absence or presence of chicoric acid was studied by MTT assay, and the membrane permeability was evaluated *via* LDH concentration assay. In Figure 6, our data revealed that oxLDL reduced cell viability and enhanced membrane permeability in HUVECs after 24 h of exposure; however, pretreatment with chicoric acid attenuated the oxLDL-induced cytotoxicity in HUVECs in a dose-dependent manner. Besides, we also confirmed the SNP (NO donor) definitely reduced oxLDL-caused cell death, confirming that eNOS function may act a key role in regulation of oxLDL-induced cell death.

### Chicoric acid reduces oxLDL-mediated p38 MAPK and NF-κ B activation

**Figure 7:**
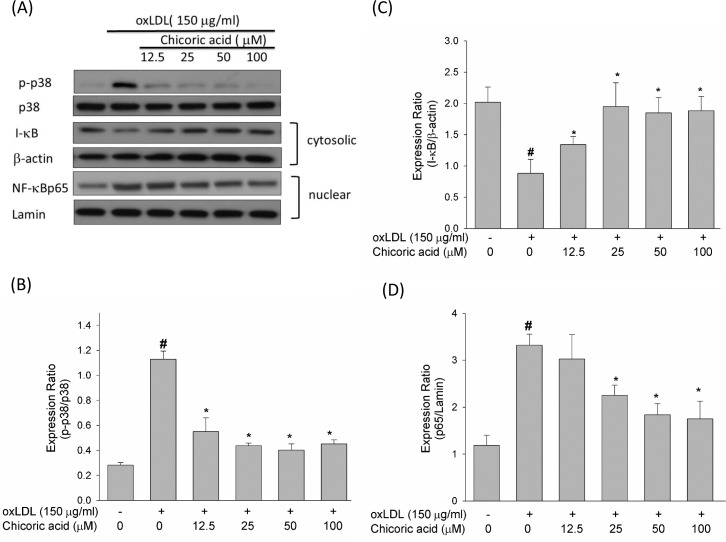
Effects of chicoric acid on phosphorylation of p38 MAPK and on the translocation of NF-kB HUVECs were pretreated with indicated concentrations of chicoric acid for 2 h followed by exposure to oxLDL (150 μg/mL) for 2 h **A**.-**D**. Western blot analysis was used to evaluate the expression of both phosphorylated and total p38 MAPK **B**., and the activation of NF-kB. Anti-β-actin and anti-Lamin antibodies were used for normalization of cytosolic and nuclear proteins **C**., **D**., respectively. The values represent means ± SD from three separate experiments. #*P* < 0.05 *vs*. untreated control; *P < 0.05 *vs*. oxLDL treatment.

The ROS produced by oxLDL were shown to up-regulate p38 MAPK and down-regulate phosphoinositide 3-kinase (PI3K). Furthermore, both of these signaling pathways lead to the activation of NF-κ B, which subsequently regulates downstream pro-inflammatory events [23]. As revealed in Figure 7A, the oxLDL promoted p38 MAPK phosphorylation was mitigated in endothelial cells pretreated with chicoric acid.

NF-κ B is a family of dimers composed of members of the Rel/NF-κ B family [24]. NF-κ B activation needs the dissociation of the repressor I-κ B. Once this dissociation occurs, NF-κ B is immediately translocated to the cell nucleus, in which it is a p65/p50 heterodimer and binds to its cognate sequence of DNA [25]. Figure 7C and 7D shown that I-κ B decreased after stimulation with oxLDL, thereby facilitating the nuclear translocation of NF-κ B p65. However, in HUVECs pretreated with chicoric acid, this phenomenon is effectively inhibited.

### Chicoric acid inhibits the oxLDL-caused attachment of THP-1 cells to endothelial cells and the activation of adhesion molecules

**Figure 8:**
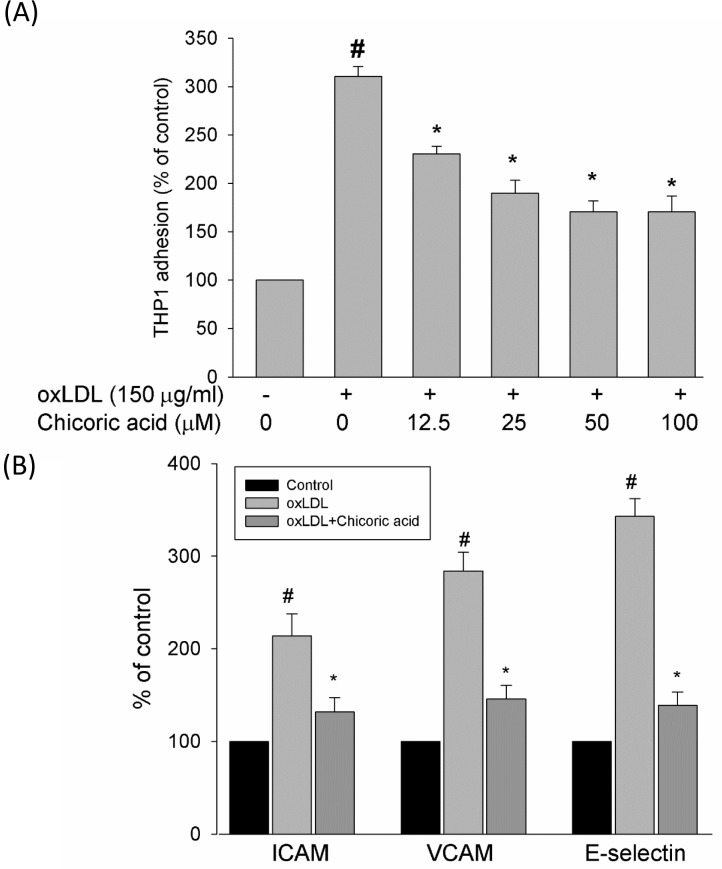
Effects of chicoric acid on oxLDL-induced adhesiveness of HUVECs to THP-1 monocytic cells and adhesion molecule expression Cells were incubated with indicated concentrations of chicoric acid for 2 h and then incubated with ox-LDL for an additional 24 h. **A**. dose-dependent effect of chicoric acid on oxLDL -induced adhesiveness of HUVECs to THP-1 was measured as described in materials and methods. HUVECs were incubated with oxLDL in the absence (control) or presence (oxLDL + chicoric acid) of 100 μM chicoric acid for 24 h. The histogram of cell surface expression of **B**. VCAM-1, **C**. ICAM-1, and **D**. E-selectin was generated by flow cytometry. (left) No treatment; (middle) oxLDL; (right) oxLDL + chicoric acid. The values represent means ± SD from three separate experiments. #*P* < 0.05 *vs*. untreated control; *P < 0.05 *vs*. oxLDL treatment.

Kim et al. reported that oxLDL-increased the retention, recruitment, and adhesiveness of human monocytes to the endothelium, a process associated with the earliest stages of atherogenesis [26]. To test the inhibitory effects of chicoric acid against monocyte adhesion to HUVECs, confluent monolayers of endothelial cells were treated with chicoric acid for 2 h and then stimulated with oxLDL for a further 24 h, followed by co-culture with THP-1 cells for 1 h in 37°C. Our results demonstrate that oxLDL increased the attachment of THP-1 cells in endothelial cells (Figure 8A). This outcome was significantly mitigated by chicoric acid intervention. The inhibitory effects of chicoric acid on adhesion molecules in endothelial cells stimulated by oxLDL were confirmed next. Figure 8B-8D, we found the expression levels of ICAM-1, VCAM-1, and E-selectin were obviously higher in endothelial cells exposed to oxLDL for 24 h than in the control samplegroup. Flow cytometry suggested that adhesion molecule expression was repressed by chicoric acid.

## DISCUSSION

It has been reported that oxidative injuries induced by oxLDL triggers the signaling pathways causing to cellular death. Furthermore, ROS formation serves as the initial pro-apoptotic signal of oxLDL, thereby activating multiple downstream pathways [1]. In our study, we have shown that chicoric acid ameliorates oxLDL-facilitated dysfunction in endothelial cells by repressing inflammatory responses and oxidative damage. Chicoric acid inhibited the production of ROS, which subsequently inhibited the function of both SOD and catalase, promoted the bioavailability of NO and maintained the mitochondrial membrane, thereby preventing pro-apoptotic responses in oxLDL-treated endothelial cells.

In normal cells, the balance between ROS formation and the activity of antioxidant enzyme is maintained [27]. There are different types of ROS produced in endothelial cells, including the hydrogen peroxide (H_2_O_2_), superoxide (·O_2_), peroxynitrite (·ONOO), hydroxyl (·OH) radicals and NO. The intracellular ROS concentration is constant as a result of the homeostasis between ROS-generating enzymes and antioxidant enzymes; however, the function of these antioxidant enzymes is inhibited by H_2_O_2_, which is produced by the dismutation of the superoxide anion [28]. Here, we confirmed that intervention with chicoric acid mitigated the oxLDL-caused inactivation of both catalase and SOD and reduced ROS formation in HUVECs stimulated with oxLDL (Figure 2). We presume that the primary mechanism through which chicoric acid mitigates oxLDL-facilitated cell death of endothelial cells is its antioxidant function.

NO plays an important role in the regulation of leukocyte adhesion, vasodilation and platelet aggregation. Inhibition of NO production and bioavailability is considered to be the primary issue in the progression of atherosclerotic lesions [29]. Moreover, the altered rate of NO production with the enhanced removal of NO causes to an obvious inhibition in the bioavailability of NO, an occurrence observed in many vascular pathologies. NO inhibits the expression of cell surface adhesion molecules, such as VCAM-1, P-selectin and ICAM-1, and prevents both the expression of MCP-1 and reduced platelet adhesion. In this article, we demonstrated that pretreatment with chicoric acid obviously restores the suppression of the eNOS protein (Figure 3) *via* oxLDL stimulation, resulting in a protective effect *via* the prevention of oxLDL-facilitated adhesion between monocytes and HUVECs and the overexpression of adhesion molecules (Figure 7) through a mechanism potentially linked to the inhibition of oxLDL-promoted ROS formation.

There is a report that activation of NF-κB is accompanied by increased P53 expression [30], which subsequently facilitates a structural change in Bax that allows mitochondrial translocation of the pro-apoptotic protein Bcl-2 and facilitates the activation of caspase 3 [31]. In our study, we found that chicoric acid inhibited the translocation of NF-κB from the cytosol to the nucleus (Figure 7), suppressed Bax and obviously promotes the Bcl-2 expression, which prevented the activation of caspase 3 and subsequent DNA strand breaks (Figure 4).

The anti-atherosclerotic function of chicoric acid was reported previously. Martin group suggested that chicoric acid inhibits pro-inflammatory cytokine-induced overexpression of adhesion molecules and adherence of monocytes. Both of these events are highly related with atherosclerosis. They also concluded that chicoric acid may be a prudent compound for prevention of cardiovascular disease [32]. The conclusion of this paper is very similar with our study, we confirmed that chicoric acid protects against oxLDL-induced adhesion molecules up-regulation and adherence of monocytes (Figure 8).

The concentrations (12.5-100 μM) used are similar to those used in studies to test the inhibition of other oxidative stress-related events. For example, 100 μM of chicoric acid has been reported to reverses insulin resistance as well as mitigates pro-inflammatory events in the glucosamine-treated HepG2 cells [33]. There is increasing interest in assessing the clinical efficacy of dietary supplements, natural extracts, antioxidants and polyphenol to improve health and prevent disease [34]. Therefore, understanding the molecular mechanisms through which a dietary antioxidant can prolong diseases is important to effectively couple these improvements with healthy lifestyle changes.

**Figure 9:**
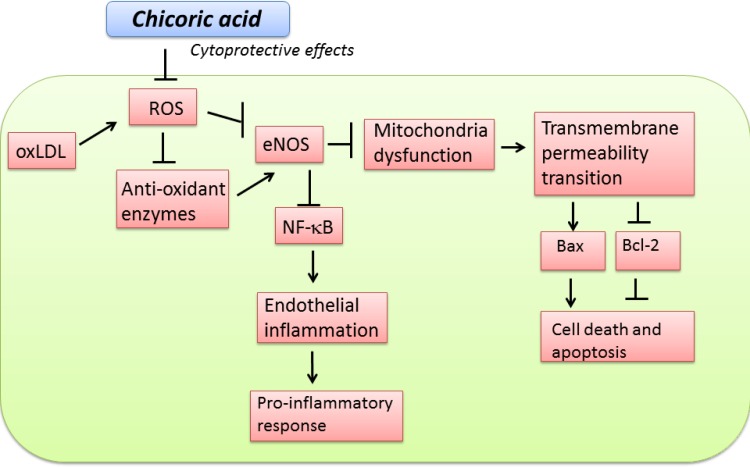
Schematic diagram showing cytoprotective signaling of chicoric acid in oxLDL-induced oxidative injuries in endothelial cells The (→)indicates activation or induction, and (┤)indicates inhibition or blockade.

In conclusion, our findings suggest that chicoric acid mitigates oxLDL-induced endothelial dysfunction and inhibits oxLDL-facilitated ROS formation and the inactivation of antioxidant enzymes. chicoric acid repressed the adhesion molecules and the adherence of monocytes by attenuating NF-κB activation. Chicoric acid treatment also inhibited oxLDL-facilitated endothelial apoptosis (Figure 9). It is likely that these beneficial effects contribute to the overall antiatherogenic function of chicoric acid. However, only *in vitro* investigations were used to tested the cytoprotective effects of chicoric acid from oxLDL-caused endothelial dysfunction. Animal study will be used to further evaluate the anti-atherosclerotic effects of chicoric acid.

## MATERIALS AND METHODS

### Reagents

Fetal bovine serum, trypsin-EDTA, and M199 were bought from Gibco (Grand Island, NY, USA); low serum growth supplement (LSGS) was bought from Cascade (Portland, OR); Chicoric acid, 2’, 7’-bis-2-carboxyethyl-5 (and -6)-carboxyfluorescein-acetoxymethyl ester (BCECF-AM), ethylene diaminotetraacetic acid (EDTA), 2’,7’-dichlorofluorescein acetoxymethyl ester (DCF-AM), 5,58,6,68-tetraethylbenzimidazolcarbocyanine iodide (JC-1), SNP, streptomycin and penicillin were bought from Sigma (St. Louis, MO); the TUNEL assay kit was bought from Boehringer Mannheim (Mannheim, Germany); the superoxide dismutase activity assay kit was bought from Calbiochem and catalase activity assay kit were bought from Abcam; active caspase 3 assay kit was obtained from BioVision (Palo Alto, CA); anti-intercellular adhesion molecules (ICAM-1), anti-vascular cell adhesion molecule-1 (VCAM-1), anti-E-selectin and the annexin V apoptosis kit were obtained from R&D Systems (Minneapolis, MN); anti-eNOS, anti-p-eNOS, anti-Bcl 2 and anti-Bax were obtained from Transduction Laboratories (CA, USA).

### Cell culture

Human umbilical vein endothelial cells (HUVECs), obtained from ATCC (ATCC® PCS-100-010), were cultured in 199 medium with a low serum growth supplement. Culture dishes were coated with gelatin for 2 hours. Penicillin and streptomycin were used as an antibiotic. Trypsin-EDTA was used for cell passage. THP-1 were cultured in RPMI with 10% FBS at a density of 2 to 5×10^6^ cells/ml, as suggested in the product specification sheet provided by the vendor.

### Lipoprotein oxidation

Human plasma LDL was purchased from Sigma. CuSO_4_ (10 μM) were used to oxidase LDL. After oxidation, CuSO_4_ was removed by PD10 columns (GE). The oxidative status of LDL were confirmed by thiobarbituric acid reactive substances (TBARS) assay.

### Immunoblotting

Endothelial cells were pretreated with chicoric acid for 2 hours, and then treated with oxLDL for 24 hours. At the end of treatment, the cytosolic/nuclear protein fractions of endothelial cells were isolated by a Cytoplasmic Extraction kit, dependent on the manufacturer's instructions (PIERCE, Rockford, IL). Total protein determined by SDS-PAGE and an immunoblot assay. The blots were incubated with 5% milk for 1 hour and then incubated with primary antibodies 1 hour at room temperature respectively, followed by incubation with horseradish peroxidase-conjugated secondary antibody for 1 hour. An enhanced chemiluminescent assay (ECL; Amersham, Berkshire, UK) was used to detect the bound immunoproteins. The intensities of protein expressions were quantified by densitometric analysis (Digital Protein DNA Imagineware, Huntington Station, NY). Protein expressions were normalized by internal control genes and indicated by bar-chart.

### Investigation of cytotoxicity and indices of apoptosis

To test the effect of chicoric acid on oxLDL-facilitated cytotoxicity, endothelial cells were treated with different concentrations of chicoric acid for 2 hours and then stimulated with 150 μg/ml oxLDL for a further 24 hours. MTT assay was used to test cell viability. Annexin V assay and the terminal deoxynucleotidyl transferase-mediated dUTP nick end-labeling (TUNEL) assay were used to determine apoptotic cells by flow cytometry.

### ROS formation assay

Endothelial cells were incubated in 96-well. Confluent endothelial cells were pre-treated with chicoric acid for 2 hours. oxLDL was then incubated to the medium with or without chicoric acid. The fluorescence microplate reader was used to analyze fluorescence intensity by an excitation at 540 nm and an emission at 590 nm. This formula [(Ft_2_-Ft_0_)/ Ft_0_]X100, was used to calculate the percentage increase of fluorescence (Ft_2_ is the fluorescence at 2 hours of oxLDL exposure and Ft0 is the fluorescence at 0 hour of oxLDL exposure).

### Measurement of antioxidant enzyme activity

Confluent HUVECs were pre-treated with chicoric acid for 2 hours. oxLDL was then incubated to the medium with or without chicoric acid. SOD and catalase activity were tested via an enzymatic assay method using a commercial kit dependent on the manufacturer's instructions.

### Expression level of adhesion molecules

Confluent HUVECs were pre-treated with chicoric acid for 2 hours. oxLDL was then incubated to the medium with or without chicoric acid. After stimulation, endothelial cells were collected and incubated with FITC-conjugated anti-body for 30 min at room temperature. The intensities of fluorescence intensity was quantified by flow cytometry.

### Investigation of mitochondrial transmembrane potential

The mitochondrial transmembrane potential (ΔΨm) were tested by JC-1. After stimulation of oxLDL for 24 hours, endothelial cells were collected and JC-1 (5 μM) was loaded for 30 min. Investigation of the ΔΨm was carried out by flow cytometer.

### Investigation of active caspase-3

To test the effects of chicoric acid on oxLDL-caused caspase-3 activation, HUVECs were pre-treated with chicoric acid for 2 hours. oxLDL was then incubated to the medium with or without chicoric acid. The status of active caspase-3 was investigated by flow cytometry using a commercial CaspGLOWTM Red Active Caspase-3 Staining Kit (BioVision).

### Monocytes adherence assay

HUVECs were pre-treated with chicoric acid for 2 hours. oxLDL was then incubated to the medium with or without chicoric acid. THP-1 cells were labeled by BCECF-AM 4 μM. At the end of stimulation, medium were removed and 0.1 mL/well of THP-1 cells were incubated for 1h. The cells were allowed to adhere at 37°C for 1 hour in a 5% CO_2_ incubator. Non-adherent cells were washed by PBS. The adherent cells were lysed by 100μl PBS with 0.25% Triton X-100. The fluorescence microplate reader was used to measure the fluorescence intensity by an 485 nm (excitation) and 538 nm (emission).

### Analyses

Results are expressed as mean ± SD. Differences between the groups were analyzed using one-way ANOVA followed by post-hoc tests. A *P*-value <0.05 was considered statistically significant.
